# The impact of aging on the spatial accuracy of quick corrective arm movements in response to sudden target displacement during reaching

**DOI:** 10.3389/fnagi.2015.00182

**Published:** 2015-09-23

**Authors:** Daisuke Kimura, Koji Kadota, Hiroshi Kinoshita

**Affiliations:** Biomechanics and Motor Control Laboratory, Graduate School of Medicine, Osaka UniversityToyonaka, Japan

**Keywords:** online feedback control, reaching accuracy, reflexive correction, implicit motor control, response latency

## Abstract

Age-related declines in visuomotor processing speed can have a large impact on motor performance in elderly individuals. Contrary to previous findings, however, recent studies revealed that elderly individuals are able to quickly react to displacement of a visual target during reaching. Here, we investigated the influence of aging on quick, corrective responses to perturbations during reaching in the terms of their functional contribution to accuracy. Elderly and young adults performed reaching movements to a visual target that could be displaced during reaching, and they were requested to move their hand to reach the final target location as quickly as possible. Results showed that, for the younger group, the variance in the directional error of the corrective response correlated with the variance in the reaching trajectory at the halfway point of the reach, but the correlation decreased at the end of the reaching. On the other hand, such correlations were not significant in elderly participants, although the variance of the directional error did not show a significant difference between age groups. Thus, the quick, corrective response seems to play an important role in decreasing variability, especially before the end of reaching, and aging can impair this process.

## Introduction

To interact appropriately with a dynamically changing environment, individuals must continuously correct their motor patterns, even after a planned movement has been initiated. Age-related deterioration in this ability, along with functional declines in the skeletomotor apparatus and sensory organs, may pose certain challenges for an elderly individual during their daily life (Goggin and Meeuwsen, 1992; Chaput and Proteau, 1996; Skoura et al., 2005; Doherty, 2003; Poston et al., 2013; Van Halewyck et al., 2014). Similarly, in the case of reaching toward a movable target, several psychological and neurophysiological studies have revealed that hand trajectory corrections become slower and more inaccurate with age (Cooke et al., 1989; Castiello et al., 1998; Sarlegna, 2006; Cheng et al., 2012). Most studies have concluded that deterioration in explicit mental processes, such as stimulus detection and volitional action planning (Chaput and Proteau, 1996; Skoura et al., 2005; Seidler et al., 2010), are the main causes for this age-related decline. To compensate for these multifactorial age-related changes in sensorimotor systems and achieve a performance level for voluntary motor tasks equivalent to that of young individuals, it is assumed that elderly persons must recruit additional and/or different brain regions. Indeed, several studies have observed greater multi-regional activation in elderly adult brains as compared to young age groups with the same performance level during the execution of online control tasks (Ward and Frackowiak, 2003; Heuninckx et al., 2008; Sharma and Baron, 2014). Sometimes, this additional cognitive processing in elderly individuals could lead to increased reaction times and movement durations (Pratt et al., 1994; Yan et al., 1998; Salthouse, 2000).

However, as demonstrated by motor control studies over the last two decades, most sensorimotor signals are processed in an implicit manner and are largely independent of explicit mental processes. For example, according to Goodale et al. (1986), correction of a hand trajectory toward a displaced target during reaching, which is referred to as the “automatic pilot” or “target jump response” (TJR), can occur without conscious control. Recent studies have demonstrated a short latency (within 150 ms in young individuals) for this response (Day and Lyon, 2000; Kadota and Gomi, 2010); additionally, elderly individuals show this response with a very small (around 20 ms) age-related prolongation under the correct conditions (Rossit and Harvey, 2008; Kadota and Gomi, 2010). Since this latency prolongation is comparable to the age-related decrement of early-stage visual processing (Fiorentini et al., 1996), it may largely be due to sensory slowing (Kadota and Gomi, 2010) and not deterioration of motor factors such as discrimination or action selection. Indeed, considering the latency, it appears that the response is induced prior to voluntary corrective responses, which are observed more than 200 ms after the onset of target movement (Yan et al., 2000; Sarlegna, 2006). This suggests that TJR processing is independent of voluntary corrections based on explicit perception of changes in the visual environment (Desmurget et al., 1997; Gomi, 2008; Gaveau et al., 2014). As a result, processing speed should be free from the effects of aging on cognition though multiple computations are involved in this process (Kadota and Gomi, 2010). If so, it is likely that not only speed but also spatial TJR accuracy, which relates to arm trajectory direction changes in response to the target movement direction, is maintained in elderly individuals. In this case, we should find TJR directions for elderly individuals to be similar to those of young individuals. It is also of interest to understand the extent to which the magnitude of TJR spatial errors relates to the magnitude of the final (endpoint) spatial errors. This could provide information on the functional contribution of TJR to neural processing for end-stage visual guidance of a hand toward a target.

Generally, an emerging movement pattern is a mixture generated by cognitive and implicit sensorimotor controls. To distinguish the latter from gross movement patterns, some studies have employed a cognitive-control reaching task. In this task, called an “anti- task,” participants are required to move their arm in the opposite direction of a target displacement direction as soon as possible; this is because the target displacement is consciously apparent during reaching, raising the issue of the degree to which conscious process contribute to the TJR (Day and Lyon, 2000; Day and Brown, 2001). In the anti-task, an early, inappropriate deviation of the reaching arm in the same direction as the target displacement occurs by implicit processes. The subsequent arm movement in the same direction as the target displacement occurs intentionally. Therefore, the onset time of the anti-direction movement can be taken to correspond to the latency of voluntary corrective movements generally. Motor responses emerging prior to the onset time can be regarded as non-intentional, implicit control components (Day and Lyon, 2000). Hence, by applying the anti-task, these motor components (purely generated by implicit processes) are supposed to be temporally separable from cognitive components. Thus, a comparative analysis of the two components would be available. It is predicted that age-related deterioration in cognitive processing would only have a minimal effect on these early components since such components seem to be largely generated by implicit processing. In contrast, the later, voluntary reaction is probably subject to the effects of cognitive aging. Therefore, the present, cross-sectional study investigated the effects of aging on spatial TJR accuracy. Age-related changes in the contribution of implicit and voluntary sensorimotor processing to online corrective movement were also assessed. First, online corrective responses induced by implicit processes during corrective reaching were determined from the onset timing of voluntary correction as measures by the anti-task. In detail, the onset timing of anti-direction response in the anti-task was defined as the onset timing of the response produced by participant volition. Next, for the pro-task, the hand velocity vector before 150 ms to the onset timing of voluntary control were calculated as the implicit component of the TJR. To evaluate spatial TJR accuracy in the pro-task, the difference in direction of the hand velocity vector relative to the target displacement direction was computed while healthy young and elderly individuals performed visual target-displacement reaching tasks. The importance of the TJR for endpoint accuracy was also examined by evaluating the relationship between the TJR directional difference and the endpoint variability, broken down by age group.

## Materials and Methods

### Participants

The participants were 25 young adults (mean age ± SD 22.6 ± 4.2 years, 12 males) and 25 elderly adults (69.6 ± 5.4 years, range 61–80 years; 13 males) who were all right-handed. Elderly participants all had a Mini Mental State Examination (MMSE) score above 28 out of a maximum of 30 points (mean score 29.9 points). None of the participants reported any motor or sensory deficits, including visual disorders, and all had normal or corrected-to-normal vision. Written informed consent was obtained from each participant, and the Research Ethics Committee at the Graduate School of Medicine, Osaka University, approved the study.

### Experimental Setup

**Figure 1:**
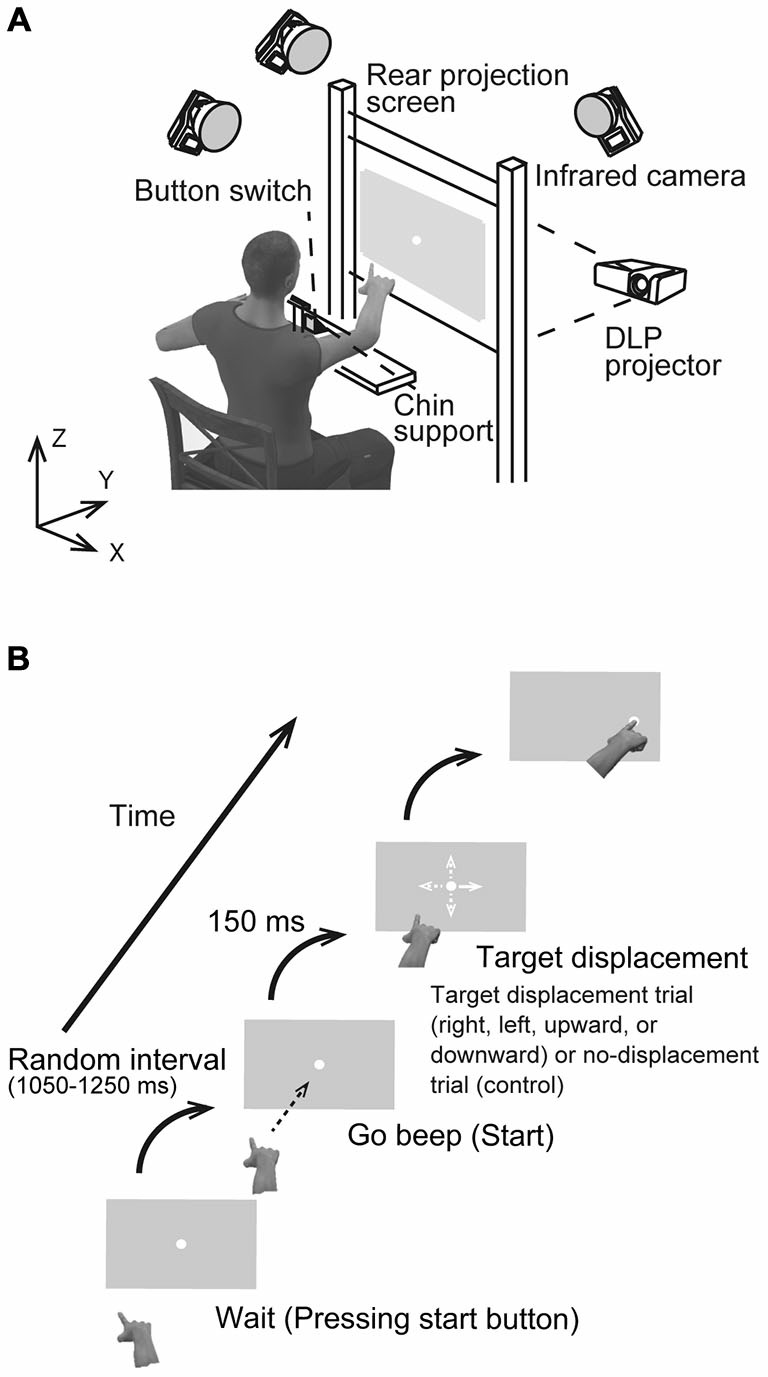
**(A)** Schematic drawing of the experimental setup. Participants were asked to reach toward a visual target presented at the center of a screen placed at a distance of 0.5 m in a darkened room. **(B)** The sequence of a visual stimulus in the rightward target displacement condition. Four possible target displacement directions were implemented: to the right, left, upward, or downward from the center (all distances 4.5 cm). In the control condition, the target remained at the central position.

Figure 1A shows the experimental set-up. A rear projection screen (760 × 560 mm), on which the visual stimulus was back-projected by a DLP projector (Pro8500, ViewSonic), was placed at a distance of 50 cm from the participant’s eyes. Visual stimuli were generated using Matlab (Math Works, Natick, MA, USA) and Cogent Graphics (University College London, London, UK) on a Microsoft Windows (Seattle, WA, USA) operating system. A photodiode was attached to the bottom left corner of the screen to detect the actual stimulus start time at a temporal resolution of 2 kHz. Each participant sat in a quasi-darkened room, and placed his/her chin on a support to stabilize the head. Using the tip of the right index finger, he/she then pressed a button switch attached to the table placed in front of the participant. The switch was connected to the computer’s parallel port, and the button release indicated onset of the reaching movement. A reflective marker was placed near the distal interphalangeal joint of the right index finger. The right wrist and index finger joints were immobilized using a plastic splint to prevent finger shaking. The marker position was recorded with three cameras located 2.5 m above the floor, which were interfaced with a motion capture system (Oqus 300, Qualisys, Sweden) working at a sampling frequency of 500 Hz. The spatial resolution was less than 0.15 mm in the mediolateral (*x*), horizontal (*y*), and vertical (*z*) directions.

### Experimental Task and Procedure

Each participant performed the task using pro- and anti-reach paradigms. In the pro-task, a reaching movement was directed towards a stimulus presented first in the center of a vertical grey screen (120 Hz refresh rate, 1024 × 768 pixel resolution) and later displaced in one of four directions (Figure 1B), On the other hand, the reaching movement was performed toward an imaginary stimulus located in the direction opposite to the target displacement direction in the anti-task. In both tasks, participants were asked to reach quickly and accurately and to touch the actual target (pro-task) or the imaginary target (anti-task) using their right index finger. The visual target was a small, filled white circle 0.8 cm in diameter. Figure 1B illustrates the sequence of events for stimulus presentation. To initiate a trial, the participant pressed the button with the right index finger and held in down. After a random interval of 1.05–1.25 s, a beep was presented to cue the participant to release the button and to reach and touch the target. The participant was not required to react immediately to the start beep to avoid any cognitive load that would affect movement stability. After a delay of 600 ms from the first beep, a second beep was presented, and the participant was required to touch the target at the time. Each participant performed two sets of 96 reaching trials, one set for the pro-task and one for the anti-task. Of the resulting 192 trials, 144 trials (75%) were TJR trials, and the remaining 48 were non-displacement controls where the target remained at the same spot (center). For the TJR trials, the target displaced to a new location 150 ms after the release of the button. By adjusting this delay, the phase when the hand trajectory is tested for TJR could be set to just before the voluntary and slower adjustment phase at the end of reaching. This strategy allows examination of the effect of the TJR on the accuracy of voluntary feedback control in a straightforward manner.

The new target location was approximately 4.5 cm to the right, left, above, or below the center, and 18 trials were performed for each of these four locations. Trial order was randomized within each task, and set order was counterbalanced across participants. Before the experimental tasks, each participant underwent a practice session in which 50 trials were performed for each of the pro- and anti-tasks. For the pro-task trials, the directional TJR differences and the inter-trial endpoint variability were evaluated. For the anti-task, the time window of the automatic corrective movement of the TJR measuring from stimulus displacement was determined.

### Data Analyses

The three-dimensional kinematic data of the index finger marker were digitized to obtain displacement data. The displacement data between 0.5 s before and 1 s after the target displacement for each trial was extracted for subsequent analysis. Each of the mediolateral (*x* direction in Figure 1A), horizontal (*y* direction), and vertical (*z* direction) displacement data were then filtered using a Butterworth low-pass filter at a cutoff frequency of 20 Hz. Velocity and acceleration data were then obtained by single and double numerical differentiation, respectively, of the displacement data for each trial. We excluded 60 of 72 TJR trials in each task type and 40 out of 48 control trials to remove outliers by means of the root mean square of the acceleration pattern difference from the corresponding median pattern. The mean of acceleration patterns for each visual stimulus condition was calculated. The evaluation period was from 100 ms before to 300 ms after the target shift.

For TJR evaluation, the time point of the maximal extent of the TJR (i.e., the endpoint of the implicit motor response) needed to be determined for each participant. To achieve this, we used results from the anti-task since the reaching finger exhibited a clear directional switching action in the frontal plane that separated the TJR from voluntary feedback action. This produced a peak TJR at the time of directional switching, as described in the Introduction. For each participant and stimulus direction, we computed the resultant acceleration curve using the mean mediolateral and vertical acceleration curves after subtracting the corresponding mean data collected during the control task. Using the mean curves, the time of zero acceleration during TJR was determined for each participant. This time was used as the peak time of his/her reflexive TJR across all stimulus directions. This time also corresponded to the time of peak velocity of the reflexive TJR, which was defined here as “peak TJR time.” The spatial accuracy of the TJR during the pro-task was evaluated using mediolateral and vertical velocity data: in other words, the rate of change in finger position in the frontal plane. A velocity vector from the time of the initial TJR (150 ms before the peak TJR time) to the peak TJR time was computed, and the angle of this vector relative to the axis of stimulus displacement direction was computed. The counterclockwise direction was defined as positive. For subsequent statistical TJR analyses, the absolute values of directional difference and inter-trial variability data were used as dependent variables.

The onset and end times of the reaching movement were determined as the moments when the horizontal velocity of the finger increased to 10 mm/s and decreased to 10 mm/s, respectively. To examine reaching precision during the pro-task, the SD values of the mediolateral and vertical finger-position components at the end time were computed. The root-mean square of the two SDs was then computed to give a single value summarizing of endpoint variability. In targeted reaching tasks generally, additional feedback correction of the hand position based on visual information about finger and target positions emerges during the final phase (Prablanc et al., 1986; Desmurget et al., 1998); this is the case even when participants are required reach the target in one step. This additional finger-position correction might override the contribution of TJR to accuracy. Therefore, the kinematic variables value were also computed at the moment when the finger velocity had decreased to 300 mm/s prior to stopping, to more directly evaluate the effect of TJR on the late phase of reaching movements.

The TJR latency (i.e., the period from the time of target displacement to the start of the arm response) was defined for each participant according to a previous method by Kadota and Gomi (2010). For this calculation, we focused on the acceleration profiles broken down by target displacement direction, that is, the *x*- and *z*-direction accelerations for the mediolateral (right- and leftward) and the vertical (up- and downward) target displacement trials, respectively. The appearance time of a significant difference between the acceleration profiles for opposite directions was detected by successive *t*-tests (*p* < 0.05) continuing for at least 20 ms in a window between 0 and 300 ms; these were the response latencies for each participant. The mean of the values calculated for the two components of motion was used as the response latency for the participant.

Endpoints greater than 3.5 SDs from the mean were considered outliers and excluded from any analyses. A mixed-design two-way ANOVA (Group × Stimulus direction) was performed on each of the dependent variables (directional TJR difference, inter-trial TJR variability, and endpoint variability). Mean values for control trials were compared with a *t*-test to detect differences between age groups. Pearson product-moment correlations were also performed to test for relationships between these dependent variables. A *p*-value less than 0.05 was considered statistically significant.

## Results

### Hand Kinematics

**Figure 2:**
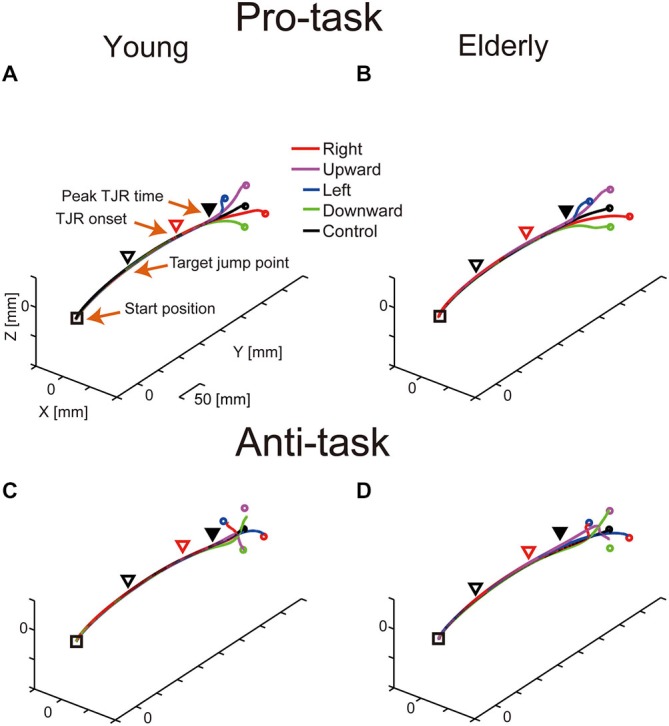
**Typical examples of mean reaching trajectories during the pro-task (A,B) and anti-task (C,D) performed by young (A,C) and elderly (B,D) participants**. Squares and triangles indicate the starting hand position and the mean hand position at the moment of target displacement, respectively. Red triangles indicate TJR onset, and filled, black triangles indicate peak TJR time. Circles represent endpoints in the pro-task for each target displacement direction.

Figure 2 shows representative mean reaching trajectories during the pro- and anti-tasks performed by young and elderly participants. For the TJR trials, the corrective reaching movement of the finger during the pro-task commonly occurred relatively close to the screen (Figures 2A,B). During the anti-task, the hand first moved towards the opposite side and then switched direction; thus, the corrective reaching movement occurred even closer to the screen compared to the pro-task (Figures 2C,D). For any of these trajectories, the early part of the TJR was hardly discernible due to a small magnitude. Furthermore, differences between young and elderly participants regarding reaching trajectories were not evident.

**Figure 3:**
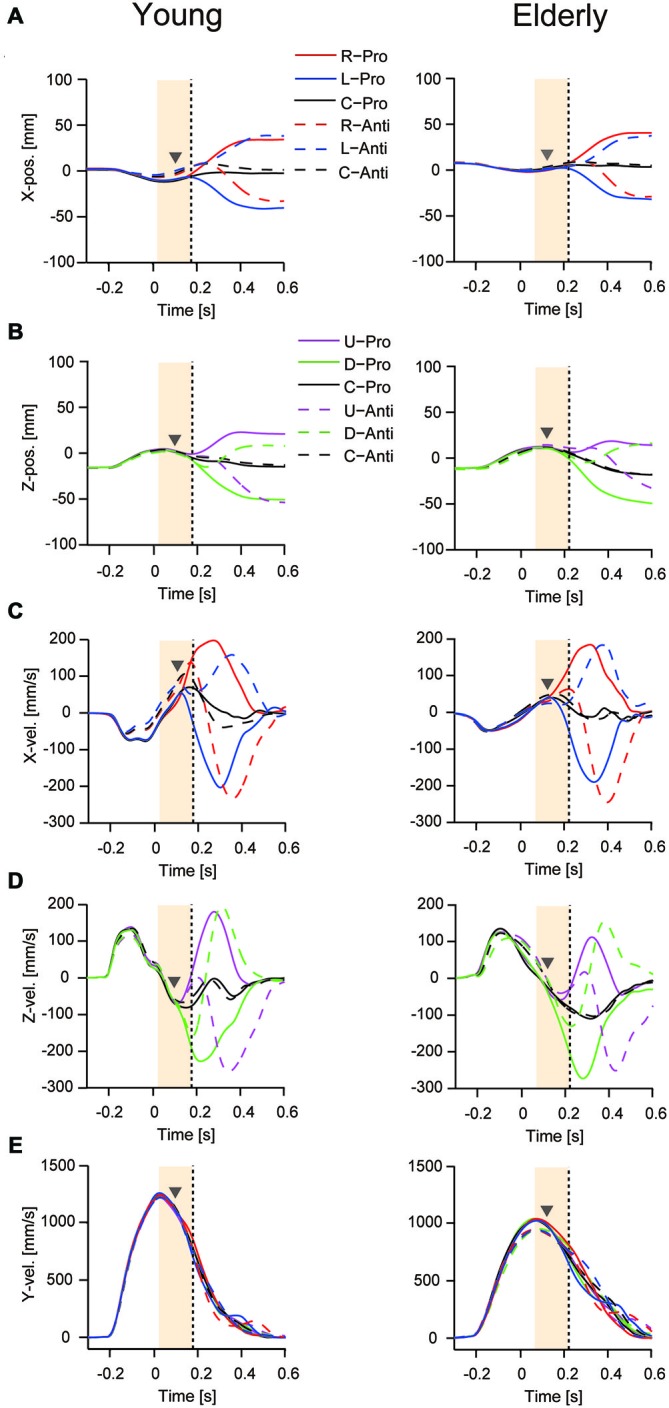
**Mean time course for reaching finger displacement and velocity in one young (left panels) and one elderly (right panels) participant during the pro- and anti-tasks**. Displacement **(A)** and velocity **(C)** for the mediolateral direction with right- and left-ward displacements. Displacement **(B)** and velocity **(D)** for the vertical direction with up- and down-ward displacements. Bottom panels are horizontal reaching movement velocities (*y*-direction) **(E)**. A time of 0 s corresponds to the onset of the target displacement to a new position. Gray triangles represent TJR onset, and vertical dots lines show peak TJR time for each participant. Orange shaded areas show a period for calculating the directional differences of TJR.

Figures 3A,C show the mean curves of mediolateral displacement and velocity for young and elderly participants when responding to right and left target displacement during the pro- and anti-tasks. Figures 3B,D show the corresponding vertical displacement and velocity for upward and downward target displacements. Figure 3E shows horizontal reaching movement velocities (*y*-direction). The TJR was not evident in any of the displacement trajectories for either young or elderly participants. In contrast, the velocity trajectories showed a notable early response, indicating a TJR at around 150 ms during the pro- and anti-tasks for the four directions. Note that during the anti-task, a TJR occurred in the direction of the target displacement, indicating that this early movement modulation was not under volitional control. The corrective movements shown in these displacement and velocity curves were quite similar for the different target-displacement directions.

The kinematic properties of the reaching movement for the young and elderly groups are shown in Table 1. Mean movement duration for the elderly group was longer than that for the young group under all target-displacement conditions. The duration ANOVA demonstrated significant main effects of both Group (*F*_(1,48)_ = 12.37, *p* < 0.0001) and Direction (*F*_(4,192)_ = 71.65, *p* < 0.0001) but a non-significant interaction (*F*_(4,192)_ = 1.63, *p* = 0.168). In contrast, there was no Group difference for the peak reaching velocity (Table 1). The ANOVA revealed neither a main effect of group (*F*_(1,48)_ = 1.589, *p* = 0.214) nor a main effect of Direction (*F*_(4,192)_ = 0.452, *p* = 0.771); the interaction was also non-significant (*F*_(4,192)_ = 0.2.157, *p* = 0.075). Additionally, the ANOVA of the intervals between reaching initiation and peak reaching velocity did not demonstrate a main effect of Group (*F*_(1,48)_ = 0.149, *p* = 0.701), or an interaction (*F*_(4,192)_ = 1.882, *p* = 0.115). On the other hand, the effect of Direction was significant (*F*_(1,192)_ = 6.630, *p* < 0.0001). *Post hoc* comparison revealed a significant difference between rightward target shift and other conditions (*p* > 0.01). However, the difference was very slight (not more than 10 ms). These results suggest that age-related slowing during a reaching movement occurred only during a later phase of reaching, and in those target-displacement trials in which the displacement was most like the overall movement.

**Table 1:** **Kinematic parameters of reaching movements for age groups**.

	Target displacement direction	Age groups	
		Younger	Elderly
Movement duration (ms)	Control	673.8 ± 43.4	726.4 ± 71.7
	Rightward	669.5 ± 40.1	722.7 ± 72.2
	Leftward	713.9 ± 44.3	767.1 ± 69.8
	Upward	691.6 ± 47.6	757.0 ± 68.3
	Downward	691.3 ± 44.1	750.1 ± 68.8
Peak velocity of reaching movement (mm/s)	Control	1173.6 ± 79.1	1128.7 ± 143.9
	Rightward	1170.7 ± 74.9	1132.7 ± 134.6
	Leftward	1167.9 ± 85.6	1134.0 ± 142.4
	Upward	1172.9 ± 83.7	1122.1 ± 143.7
	Downward	1167.0 ± 82.7	1132.8 ± 134.0
Time of peak velocity of reaching movement (ms)	Control	274.0 ± 38.3	270.7 ± 32.2
	Rightward	279.6 ± 37.1	277.5 ± 39.9
	Leftward	275.1 ± 38.7	269.1 ± 31.5
	Upward	274.9 ± 39.1	274.5 ± 35.2
	Downward	276.3 ± 40.5	268.7 ± 30.6
Endpoint absolute error (mm)	Control	19.7 ± 7.3	20.2 ± 5.8
	Rightward	20.8 ± 8.1	20.2 ± 6.0
	Leftward	19.5 ± 5.5	21.5 ± 6.3
	Upward	20.1 ± 7.6	18.9 ± 5.6
	Downward	20.7 ± 7.7	21.1 ± 6.1

*n = 25 for each group*.

For both the young and elderly groups, TJR for the pro-task was induced with short latencies (108.2 ± 9.8 ms for young and 127.8 ± 16.0 ms for elderly groups; Table 2). In addition, the latency for the anti-task was short (112.8 ± 17.9 ms for young and 141.6 ± 25.6 ms for elderly groups). The ANOVA demonstrated significant main effects of Group (*F*_(1,48)_ = 30.65, *p* < 0.0001) and Direction (*F*_(1,48)_ = 12.62, *p* = 0.0009) but a non-significant interaction. The age-related increases in mean latency were only 19.6 ms and 28.8 ms for the pro- and anti-tasks, respectively, which were closely comparable to previous results (see Kadota and Gomi, 2010).

**Table 2:** **TJR latency for pro- and anti-tasks**.

	Age groups	
	Younger	Elderly
Pro-task (ms)	108.2 ± 8.1	127.8 ± 16.0
Anti-task (ms)	112.8 ± 17.9	141.6 ± 25.6
Peak TJR time (ms)	180.5 ± 15.6	221.6 ± 22.6

*n = 25 for each group*.

The peak TJR time (i.e., the onset time for a voluntary anti-direction response) demonstrated an obvious age-related increase (*t*_(48)_ = 7.507, *p* < 0.0001; Table 2). The mean value for the young group (180.5 ± 15.6 ms) was less than that for the elderly group (221.6 ± 22.6 ms). This difference (over 70 ms) suggests an initiation delay for voluntary correction away from target displacement.

### Variability of Directional Differences in TJR and Reaching Movements

Contrary to previous studies, the absolute error of the final reaching location did not demonstrate an age-related increase (Table 1), and all of the error values were smaller than results reported in previous studies (Cooke et al., 1989; Castiello et al., 1998; Yan et al., 2000; Sarlegna, 2006; Cheng et al., 2012). This could be caused by a more extensive correction of the hand trajectory during the final phase of reaching than in previous studies. Because movement initiation time was somewhat flexible, the participants were able to make repeated corrections of the final hand location based on visual feedback information, although they were required to make reaching a “one-shot” action as much as possible. In consequence, the duration of the late phase of reaching was prolonged, especially for the elderly group as described above. Thus, the elderly participants were likely to attain a sufficient endpoint accurately by spending a longer time reaching than the younger participants, thereby masking the differences in sensorimotor control ability between age groups. The results of ANOVA support this interpretation. A main effect of Direction was significant (*F*_(4,192)_ = 11.712, *p* < 0.0001), although neither Group (*F*_(1,48)_ = 2.489, *p* = 0.121) nor interaction were significant (*F*_(4,192)_ = 1.339, *p* = 0.257).

**Figure 4:**
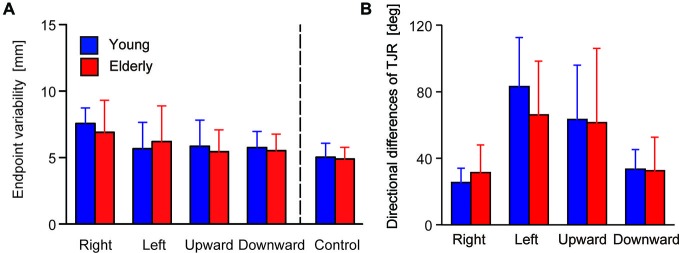
**(A)** Group means for endpoint variability. The error bars denote SD. **(B)** Median of the directional differences for all stimulus directions broken down by group.

Figure 4A shows the mean of the variability of the final reaching location, indicating a spatial consistency in reaching performance. ANOVA revealed neither a significant main effect of Group (*F*_(1,48)_ = 0.241, *p* = 0.626) nor of the interaction (*F*_(1,48)_ = 1.54, *p* = 0.192), but the main effect of Direction was significant (*F*_(4,192)_ = 21.29, *p* < 0.0001). *Post hoc* comparison revealed that variability under the rightward target-displacement condition was significantly larger than under all other conditions (leftward: *p* < 0.0001, upward: *p* = 0.002, downward: *p* < 0.0001, control: *p* < 0.0001). The upward condition showed larger variability than that of the control condition (*p* = 0.039).

The TJR directional error demonstrated different biases between mediolateral and vertical target-displacement conditions. For the younger group, the mean TJR directional error for mediolateral target-displacement conditions was relatively small (right- and leftward: −3.8 and 13.3 degrees, respectively). Compare with this, those under vertical conditions were very large (up- and downward: −25.7 and 20.6 degrees, respectively), meaning that direction accuracy of the TJR is biased laterally in this group. This tendency was weaker in the elderly group; mean values under right- and leftward target-displacement conditions were −4.3 and 10.9 degrees, respectively, and under up- and downward conditions were −15.6 and 8.2 degrees, respectively. However, more noteworthy was the magnitude of the TJR directional error. Figure 4B displays the median absolute TJR directional differences for each of the stimulus directions for the two groups during the pro-task. For most stimulus directions, the directional difference was large, except for the rightward displacements, which showed smaller directional difference than the other directions in both groups. ANOVA revealed a significant main effect of Direction (*F*_(3,144)_ = 40.267, *p* < 0.0001) but neither a main effect of Group (*F*_(1,48)_ = 0.536, *p* = 0.468) nor an interaction between Group and Direction (*F*_(3,144)_ = 1.866, *p* = 0.138). *Post hoc* comparison among conditions revealed that the variance under the rightward condition was smaller than that under left- (*p* < 0.0001) and upward conditions (*p* < 0.0001). In addition, the variance under the downward condition was smaller than that of the left- (*p* < 0.0001) and upward (*p* < 0.0001) conditions. Overall, the variances seem to be too large to permit accurate corrections of the hand trajectory toward a displaced target location; moreover, the variance may mask the effects of aging on the TJR direction.

### Relationship Between TJR Directional Variability and the Endpoint Variability

**Figure 5:**
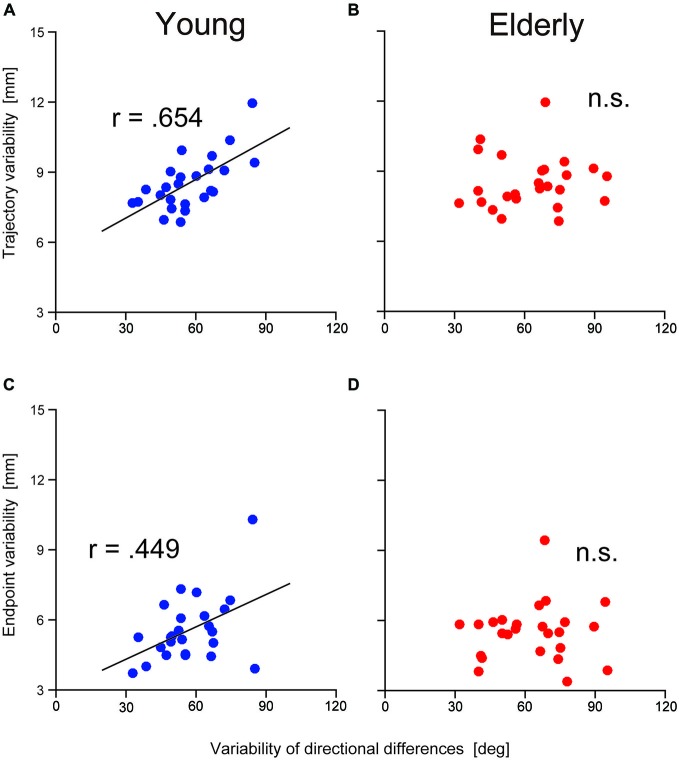
**Relationship between the variability in trajectory (i.e., hand marker location at the time when *Y*-velocity had decreased to 300 mm/s) and the variability in the directional differences for young (A) and elderly (B) participants**. Relationship between the variability in the endpoint and the variability in the directional differences for young **(C)** and elderly **(D)** participants.

To examine the contribution of TJR on reaching movement accuracy, relationships between the variability of the TJR directional difference and the spatial variability of the trajectory were examined at both of the halfway point and at the end of the reaching movements. Figures 5A,C show the relationships in the younger group. We found a strong relationship at the halfway point (*r* = 0.654, *p* = 0.0004; Figure 5A) but a weaker one at the end of reaching movements (*r* = 0.449, *p* = 0.002; Figure 5C). It appears that the variance of the reaching trajectory can affect the trajectory bias caused by TJR until a late phase of reaching, but that this variance is reduced at the fine tuning phase at the end of reaching. Thus, it seems that TJR can contribute a reduced trajectory variability, which is caused by an online corrective movement to compensate a target location change.

It is noteworthy that the above relationships were not observed in the elderly group. As shown in Figures 5B,D, correlational analysis did not reveal any significant relationships at either reaching phases. This means that the functional contribution of TJR on online correction of reaching seems to be impaired by aging although we found no age-group difference in the magnitude of variance as mentioned above.

## Discussion

The major findings from the present study were that the mean direction and inter-trial directional variability (SD) of TJR-induced hand trajectories were not affected by aging. On the other hand, the endpoint spatial variability of hand location was clearly affected by aging. Furthermore, the directional TJR variability was correlated with endpoint variability in young but not elderly participants. It seems that the TJR can contribute to maintaining reaching trajectory stability at the halfway point, and that this function deteriorates with age.

### Spatial TJR Accuracy

The SD range for the TJR directional differences was 16.1–37.8 degrees (Figure 4). These values are considerably larger than those of previous studies evaluated at longer-latencies of over 200 ms (Johnson et al., 2002; Johnson and Haggard, 2005; Sarlegna and Blouin, 2010; Brière and Proteau, 2011; Saijo and Gomi, 2012). This indicates that spatial TJR accuracy was too low to produce fine hand trajectory control. The most plausible reason for this large spatial variability seems to be a time constraint for triggering the motor response to a moving target. Considering the response latency and neural transmission time, visual information needed to produce TJR is only available during the first few 10 ms after target displacement, even if the image of a displaced target stays visible. According to psychological studies on visual motion perception, the visual motion signal during this period may be too small to accurately reconstruct information regarding motion direction. For example, Bennett et al. (2007) demonstrated that, for both young and elderly participants, motion direction discrimination decreased remarkably if stimulus presentation duration was reduced below 100 ms; at the same time, age-group differences also decreased. Additionally, the visual motion signal might have been insufficient to more accurately discriminate TJR direction in both young and elderly individuals. Considering that TJR can be induced even by a weak motion signal without a large latency increase (Veerman et al., 2008), the visual motion threshold for TJR execution may be set at a lower level than that for accurately constructing directional information.

Another reason for the large directional errors might be the low accuracy of monitoring one’s arm state during the ongoing movement. For creating a TJR within a short latency, the current hand position is estimated by comparing sensory feedback with forward model predictions to minimize delay times associated with the sensory feedback loop (Kawato, 1999; Desmurget and Grafton, 2000; Sabes, 2000). According to this computational model, forward-model estimation accuracy can be affected by sensory feedback signal accuracy and efferent copy of the motor command. Various lines of evidence suggest that an efferent copy is not sufficient for accurately localizing hand position (Bagesteiro et al., 2006; Sarlegna et al., 2006; Gritsenko et al., 2007; Medina et al., 2010). This suggests that sensory-motor responses induced with a short latency (at around 150 ms) employ information based on models using less accurate estimates. Thus, our results regarding directional TJR differences may reflect a limitation on the accuracy of quick, online movement correction.

### A Possible TJR Function

Although TJR directions were not highly congruent with target displacement directions, their variance correlated with that of endpoints in the young group (Figure 5A). This suggests that TJR functionally contributes to an accurate reaching movement trajectory. As mentioned above, TJR can be reflexively induced prior to any voluntary response. Thus, TJR functions as a coarse adjustment mechanism for reaching trajectories at the halfway point and not for fine-tuning final reach endpoints. Even this adjustment is not sufficient to completely correct the spatial deviation between current and corrected trajectories toward the displaced target location but it can instantaneously reduce the error after target displacement. This may alleviate the subsequent processing load for fine spatial adjustment, which is controlled by a long feedback loop. This speculation is consistent with previous results, which demonstrated that visual information about the hand is of little use for correcting hand movements during the initial phase of visually guided reaching (Bédard and Proteau, 2004; Dimitriou et al., 2013). In elderly individuals, voluntary corrections after TJR are inadequately generated by inaccurate motor commands due to age-related deterioration in perceptual-motor performance. Consequently, hand location variability at the end of the reaching movement may be amplified (Fradet et al., 2008; Van Halewyck et al., 2014), thus disrupting the correlation between endpoint and TJR directional variability in the elderly group (Figure 5B).

### Robustness of TJR Processes Against Aging

Another open question that needs to be addressed is why directional variability of TJR is hardly affected by aging. The neural substrates of TJR have been investigated in both psychophysical and neurophysiological studies (Desmurget et al., 1999; Desmurget and Grafton, 2000; Pisella et al., 2000). Visual motion information from the retina is progressively processed in the cortex by the dorsal motion processing pathway, including the higher extrastriate areas. From there, the information is transformed for use by the visuomotor system in the contralateral posterior parietal cortex (Good et al., 2001; Resnick et al., 2003). These and other studies have revealed age-related atrophy or neurodegeneration of brain regions forming this particular pathway (Wu and Hallett, 2005). This has been suggested as a major cause of deterioration in elderly individuals’ perceptual abilities for visual motion (Spear, 1993; O’Connor et al., 2010; Owsley, 2011). Along with this, aging has also been speculated to adversely affect the TJR, as suggested by the findings of previous studies of online control in the elderly (Castiello et al., 1998; Sarlegna, 2006; Cheng et al., 2012; Van Halewyck et al., 2014). However, our results seem to contradict these findings.

One possible explanation could be that the specific visuomotor computations for the production of TJR occurred within a very short latency. As discussed above, to quickly generate the TJR, a fast feedback loop using a predictive forward model for the reaching movement is needed (Kawato, 1999; Desmurget and Grafton, 2000; Sabes, 2000; Rossetti et al., 2003). This model assumes that motor commands for corrective movements can be generated by directly comparing the predicted and sensory-based estimates. This neuromuscular process seems to be considerably simpler than that of voluntary error correction, which imposes a heavy computational load to handle the cognitive locational comparison between the hand and target (Prablanc and Martin, 1992; Desmurget et al., 1999; Gaveau et al., 2014). The TJR process may escape the effects of age-related functional declines in complex processing, including cognition, decision making, and voluntary motor planning and execution. Therefore, considering previous results regarding response latency (Kadota and Gomi, 2010), the neural processes necessary for TJR may be preserved even in elderly individuals. Recent studies have suggested an alternative computational model emphasizing the significance of processes by the peripheral somatosensory feedback system for online movement correction (Friston et al., 2010; Friston, 2011; Adams et al., 2012, 2013). In this inclusive model, called an active inference model, motor commands for corrective movements are assumed to be mostly calculated by the spinal reflex arc constituting muscle spindles, Golgi tendon organs, and articular and cutaneous receptors. This suggests that higher cognitive processes may not contribute much to corrective movement generation. Hence, this peripheral processing system serves to generate quick, corrective motor responses to external perturbations, and may be maintained throughout the lifespan.

Another possibility for achieving normal TJR generation among elderly individuals is a re-adaptation of sensorimotor systems to compensate for the age-related decline in neural functioning, with plastic changes occurring mainly in central neural networks. A present, two main mechanisms have been proposed to explain how an elderly brain could have the capacity to generate normal performance. The first is called the “compensation hypothesis” (see review by Bernard and Seidler, 2014), which suggests that the sensorimotor-related network becomes more active and/or additional regions not observed in young individuals are recruited to maintain performance (Ward and Frackowiak, 2003; Heuninckx et al., 2005, 2008; Reuter-Lorenz and Cappell, 2008). Indeed, numerous studies have demonstrated that activity in specific regions are correlated with performance on sensorimotor tasks (Ward and Frackowiak, 2003). Therefore, in the case of online control of reaching movements, elderly individuals may also require additional neural processing to maintain a performance level similar to that of young individuals. It should be noted here that the correlation was observed not only in somatosensory- and motor-related regions but also in frontal regions that are closely related to higher cognitive processes. Furthermore, this phenomenon has mainly been documented during an explicitly-controlled motor task such as a force-grating task based on visual feedback (Ward and Frackowiak, 2003), complex inter-limb coordination movement (Heuninckx et al., 2005, 2008), and repetitive finger tapping (Shimoyama et al., 1990; Sharma and Baron, 2014). If this compensatory neural function contributes to the online control of corrective reaching in elderly individuals, it seems likely that the voluntary control process of a reaching hand during the later reaching phase (and/or its previous processing of motor planning) would benefit from this function rather than the generation of an implicit, quick response (i.e., the TJR). However, as mentioned in the introduction, the TJR appears to be generated by a unique process involving the visual dorsal stream. Considering the latency, the initial TJR signal must be transferred to the primary motor area via few synaptic relays. Hence, this process appears to be largely independent of other higher sensorimotor and cognitive processes and may be the shortest pathway for generating a visuomotor response. Therefore, it is less useful to defend the compensatory-processing. In line with this speculation, the second hypothesis regarding neural compensation (the dedifferentiation hypothesis) may also be useful (Roski et al., 2013; Sleimen-Malkoun et al., 2014; Lee et al., 2015). In this hypothesis, age-related deterioration in neural processing can be compensated by re-organization of the functional connections between brain regions. Thus, it is necessary to reveal a neural network that can engage in processing at the same latency as the originally proposed TJR circuit, but through pathways capable of age-dependent re-organization. However, evidence of such a system is lacking. Further research is needed to determine which mechanisms are adequate for maintaining neural TJR processing in elderly individuals.

In contrast to the variability of the TJR directional difference, endpoint variability showed an age-related increase. This suggests that online control occurring after the target displacement response is affected by neural aging, unlike the processes mentioned above. One of the most promising explanations for this phenomenon is the effect of aging on oculomotor function. In the case of reaching toward a visual target, which shifts in a stepwise manner, participants made saccadic eye movements toward the displaced target location and continued fixating on the target until reach termination (Prablanc and Martin, 1992; Day and Brown, 2001; Gaveau et al., 2008). Studies have confirmed that saccadic dynamics (i.e., latency, accuracy, and velocity) show age-related deterioration (Munoz et al., 1998; Irving et al., 2006); this may adversely affect the updating of visuomotor information regarding the internal representation of arm and/or target location during the final reaching phase (Henriques et al., 2002). This perturbed representation of target location may be the source of endpoint variability in elderly individuals.

Overall, the increase in reaching trajectory variability among elderly individuals, which has been reported in previous studies (Ghilardi et al., 2000; Sarlegna, 2006), could reflect deterioration in voluntary motor control associated with aging but not due to decrements in quick reflexive responses (e.g., the TJR). Further research is necessary to a thorough understanding of how aging influences the interaction between voluntary and reflexive online motor control, which could be relevant for improving elderly individuals’ daily functioning.

## Conflict of Interest Statement

The authors declare that the research was conducted in the absence of any commercial or financial relationships that could be construed as a potential conflict of interest.
